# YY1's DNA-Binding motifs in mammalian olfactory receptor genes

**DOI:** 10.1186/1471-2164-10-576

**Published:** 2009-12-03

**Authors:** Christopher D Faulk, Joomyeong Kim

**Affiliations:** 1 Department of Biological Sciences, Louisiana State University, Baton Rouge, LA 70803, USA

## Abstract

**Background:**

YY1 is an epigenetic regulator for a large number of mammalian genes. While performing genome-wide YY1 binding motif searches, we discovered that the olfactory receptor (OLFR) genes have an unusual cluster of YY1 binding sites within their coding regions. The statistical significance of this observation was further analyzed.

**Results:**

About 45% of the olfactory genes in the mouse have a range of 4-8 YY1 binding sites within their respective 1 kb coding regions. Statistical analyses indicate that this enrichment of YY1 motifs has likely been driven by unknown selection pressures at the DNA level, but not serendipitously by some peptides enriched within the OLFR genes. Similar patterns are also detected in the OLFR genes of all mammals analyzed, but not in the OLFR genes of the fish lineage, suggesting a mammal-specific phenomenon.

**Conclusion:**

YY1, or YY1-related transcription factors, may help regulate olfactory receptor genes. Furthermore, the protein-coding regions of vertebrate genes can contain *cis*-regulatory elements for transcription factor binding as well as codons.

## Background

The transcription factor YY1 is a *Gli-Kruppel *type zinc finger protein that is highly conserved from insects through vertebrates [1]. YY1 can function as an activator, repressor, or initiator depending upon the other regulatory elements in the region [2]. YY1 also interacts with a variety of proteins including components of RNA polymerase II complex, transcription factors, and histone-modifying complexes [3-5]. According to genome-wide surveys, about 10% of all human genes contain YY1 binding motifs in their promoter regions [6]. Functionally, YY1 is involved in many biological processes, including embryonic development, cell cycle progression, apoptosis, B cell development, Polycomb group Gene (PcG)-mediated repression, genomic imprinting, and X chromosomal inactivation [2,3,7]. YY1 was also initially identified as a factor controlling the transcriptional activity of the murine retrotransposon 'Intracisternal A Particle' [8]. Since then, many retroposons, including SINE, LINE, and endogenous retrovirus families, have been shown to contain YY1 binding sites in their promoter regions [3,4]. Due to this ubiquitous presence of YY1 binding sites in genome-wide repeats, YY1 has also been regarded as a surveillance gene that is responsible for repressing transcriptional background noise from these repeats [9].

The olfactory receptor (OLFR) genes of mammals encode short, single coding exon, G protein-coupled receptors that are responsible for sensing a large number of air-borne scents [10]. This gene family is comprised of over 800 and 1,300 gene members in human and mouse respectively, forming the largest gene family in mammalian genomes [10-13]. The aquatic vertebrates, the teleost fish lineage, also have a similar odorant receptor gene family [14]. However, the odorant receptor (OR) family of the fish lineage consists of a much smaller number of genes than that of the mammals, and these OR genes are also much more diverse in sequence identity than those of mammals. Mammalian OLFR genes are divided into Class I and Class II groups based on sequence identity [15]. Class II genes make up ~90% of OLFRs and are thought to have expanded during the transition to land-based living.

In mammals these olfactory receptors presumably expanded due to the selective advantage conferred by a well developed sense of smell [15]. While mice and other mammals retain function and expression of almost all OLFRs, the majority of these are pseudogenized in humans [16]. The mammalian OLFR genes are highly tissue-specific and are expressed primarily in the olfactory epithelium though a subset expresses in a chemosensory role in other tissues such as kidney and sperm [17-19]. Furthermore, only one copy (allele) out of all 1,000 OLFR genes is selected and expressed in each neuron cell of the olfactory tissue [20]. The unusual transcriptional control of the OLFR gene family is likely mediated through unknown *trans*-acting factors [21]. The tissue-specific nature of their expression coupled with their widespread duplication requires a mitotically-stable global silencing mechanism in all cell types. According to recent studies, potential *cis*-regulatory elements recruiting these *trans *factors are hypothesized to be located within the protein-coding regions of the OLFR genes rather than their surrounding genomic regions [22,23].

While performing genome-wide searches of the DNA-binding motifs of YY1, we discovered that the mammalian OLFR genes contain unusual clusters of YY1 binding sites within their protein-coding regions, whereas most YY1 binding sites are solitary and upstream of a regulated gene. In this study, we further analyzed the significance of this discovery with several bioinformatic and statistical measures, which will be described below. Specifically we test whether the presence of the YY1 binding sites could be explained by DNA sequence or amino acid motif conservation.

## Results

### YY1 DNA-binding motifs in the mammalian OLFR genes

**Figure 1:**
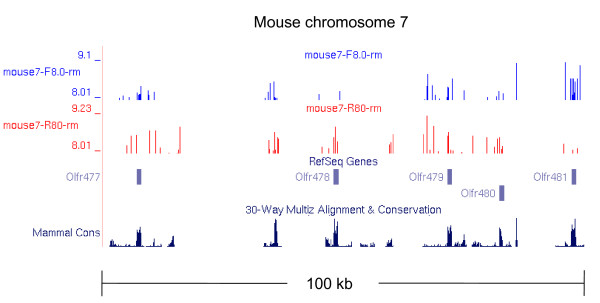
**YY1 binding sites in genomic context**. YY1 binding sites are mapped onto the Custom Track of the UCSC Genome Browser http://genome.ucsc.edu/. YY1 binding sites are marked by blue and red bars indicating forward and reverse directions respectively. Height on the Y-axis represents the score of each YY1 binding site in relation to the consensus sequence. This 100 kb segment of the genome contains five olfactory receptor genes and shows association of several YY1 binding sites correlated with the exons of the genes. Mammalian conservation is shown in the plot at the bottom of the map. This map was created through scanning the RepeatMasked sequence with a scoring matrix representing the YY1 binding site consensus. Mammalian conservation correlates with exons as expected, but also with many YY1 binding sites in this region.

YY1 is predicted to be a global epigenetic regulator based on its ubiquitous expression and interaction with many histone-modifying enzymes [4]. As part of the efforts exploring this possibility, we have performed several series of YY1 binding motif searches using the genome sequences of mammals (human, mouse, and cow) [24-26]. Here we first scanned the genome sequences of human and mouse using a Position Weight Matrix (PWM)-based Perl script. Repetitive elements are known to contain YY1 binding sites so the RepeatMasked genome was used [27]. Later, the results of these searches were visualized using the repeat-masked Custom Track of the UCSC genome browser [28]. While inspecting global localization patterns of YY1 binding motifs in each genome, we noticed that clusters of YY1 binding motifs are co-localized with the genomic regions harboring olfactory receptor (OLFR) genes. The mammalian OLFR genes show a single coding exon structure, and they are also localized as gene clusters in specific regions of mammalian chromosomes. One such example is shown using the 100-kb genomic region from Mmu 7 chromosome (Figure 1). This figure shows a representative sample of 5 OLFR genes, and the locations of these genes coincide with those of YY1 binding motifs. Each OLFR gene appears to contain a range of 4 to 8 YY1 binding motifs within its 1-kb-long Open Reading Frame (ORF). As expected, the coding regions correlate well to the placental mammal conservation plot, a default track available on the UCSC genome browser. Also, the identified YY1 binding motifs appear to be random in location within the coding regions but do show a bias in orientation with respect to the direction of OLFR gene transcription, which will be described later.

**Figure 2:**
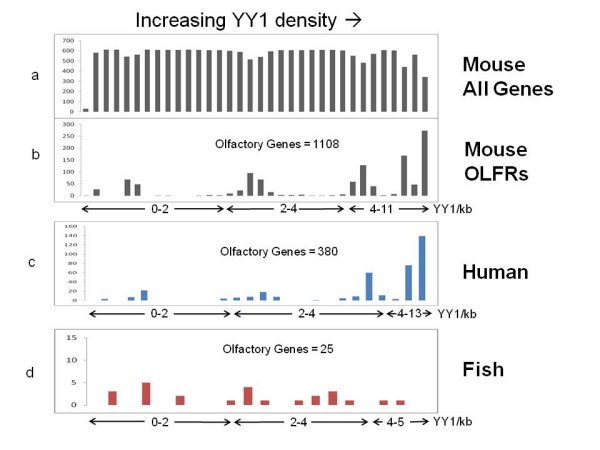
**Histogram of genes with increasing YY1 density**. Each olfactory receptor gene was scanned for the number of YY1 binding sites found within its transcript. The combined score was divided by the gene length, resulting in the YY1 density for the given olfactory receptor gene. Note that the quality of the YY1 scores varies based on the match generated by the Position Weight Matrix, and thus the calculation of YY1 binding sites per 1 kb is approximate. Genes were ordered based on increasing YY1 density and the histograms were derived by the relative position of a gene within the group of all genes. A) The mouse non-olfactory genes binned by relative order of increasing YY1 density (x-axis). The y-axis indicates the number of genes in each bin. B) Mouse olfactory genes binned in the same fashion, also from their position within the group of all genes. The highest group of bins corresponds to a density of 4-11 YY1 binding sites per 1 kb transcript for mouse though only 6 genes show a YY1 density corresponding to more than 8 YY1 sites per kb. For human and mouse (panels B&C), there is a large group of olfactory receptor genes with high YY1 density, whereas fish (D) does not show this pattern.

The unusual clustering of YY1 binding motifs within the protein-coding regions of the OLFR genes was further analyzed to test if this pattern is unique to only the OLFR genes or also found in the ORFs of other genes. For this analysis, the entire set of the mouse mRNA database was scanned with the PWM-based Perl program to identify YY1 binding motifs. The number of the identified YY1 binding motifs within a given DNA sequence corresponding to the transcript was further divided by the size of the mRNA sequence, yielding a YY1 density score. The mouse mRNA sequences (total number = 20,191) were subsequently binned based on their relative YY1 density scores (the X-axis on Figure 2a). The Y-axes of figure 2 represents the number of genes within a given range of the YY1 density score. This analysis indicated that the majority of the non-olfactory mouse mRNA sequences (19,083 sequences) were distributed evenly and randomly throughout the varying ranges of the YY1 density score (0 to 0.114) (Figure 2a). In contrast, our detailed inspection revealed that about half of the OLFR gene set (1,108) show very high YY1 density scores. To better visualize this unusual pattern, we separated only the OLFR gene set from the rest of the mRNA set, and derived another histogram (Figure 2b). As shown in Figure 2b, about 45% (or 496 of 1,108) of the OLFR genes have YY1 density scores ranging from 0.032 to 0.094, which are equivalent to 4 to 8 (or more) YY1 binding sites per a 1-kb mRNA sequence. In contrast, a scan of the 1 kb upstream regions of 20,419 Refseq genes revealed that only 10% of these putative promoter containing regions had a density greater than 4 YY1 binding sites.

We also repeated the above analyses using the mRNA data sets derived from human, cow, and zebrafish transcriptomes to test the evolutionary conservation of this unusual clustering of YY1 binding motifs within OLFR genes (Figure 2c &2d). The results from human and cow (data not shown) mRNA data sets also showed a pattern consistent with the mouse data set: an unusual clustering of YY1 binding motifs within many OLFR genes. In humans we found 57% (or 215 of 380) of OLFRs contain more than 4 YY1 binding sites while percentage in cow rose to 82% (or 740 of 900). It is important to note that the total number of the human OLFR genes (380) in the figure is smaller than those of the other mammals since a large fraction of the human OLFR genes are known to have become pseudogenes in recent evolutionary times and we removed all genes annotated as hypothetical. In contrast, the OLFR genes of zebrafish do not show a similar pattern to mammals. The total 25 OLFR genes of zebrafish show as wide a range of the YY1 density scores as seen in the other non-OLFR genes (Figure 2d). These results confirm that the unusual clustering of YY1 binding motifs within the OLFR genes is a feature of the mammalian lineage.

### Statistical tests for the clustering of YY1 binding motifs within OLFR genes

**Figure 3:**
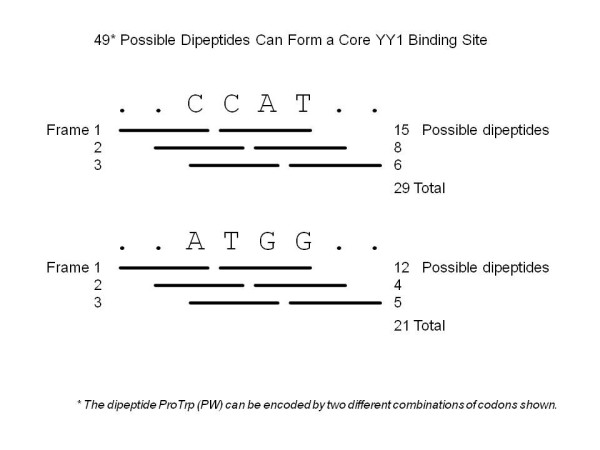
**YY1 DNA-binding motifs as translated dipeptides**. The four base pair core motif of the YY1 binding site is CCAT (forward) and ATGG (reverse). In principle, this core can code for two codons in each of the three frames. In the forward direction, CCAT can encode 29 possible dipeptides while in the reverse direction ATGG can encode 21 dipeptides, with one duplicate. These 49 dipeptides are a subset of the 400 possible dipeptides which can be found in a protein (20 total amino acids)^2^. The distribution frequency of these dipeptides has been examined to determine if any of the 49 YY1 binding sites is overrepresented in olfactory receptor genes.

**Figure 4:**
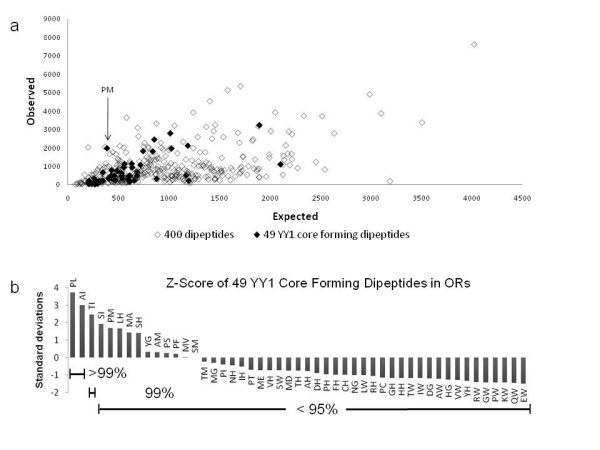
**Correlation of observed and expected dipeptides and statistical significance**. The mouse proteome was divided into olfactory genes (1,178) and all other genes (33,788) and the frequency of each dipeptide was tabulated. Of the possible 400 dipeptides, 49 can encode the core of the YY1 binding site. A) Observed vs. expected plot of 400 dipeptides (hollow diamonds) as compared to the subset of 49 YY1 binding site core forming dipeptides (filled diamonds). The subset does not deviate from the overall pattern. Pro-Met is indicated as having a much larger observed than expected count. B) Z-score of the observed and expected counts of the subset of YY1 binding site core forming dipeptides. All but three fall within 1 standard deviation of their expected values. Only Ala-Ile, and Pro-Leu deviate significantly from their expected values.

We performed two different series of analyses to test the functional significance of the observed clustering of YY1 binding motifs within the mammalian OLFR genes. First, we tested whether some peptide motifs enriched within the protein sequences of the OLFR genes are responsible for a spurious display of the YY1 binding motifs in the nucleotide sequences of the OLFR genes. If some peptide motifs are enriched then we expect a significant difference in the number of those motifs found in OLFR genes versus the rest of the proteome. For this test, we identified 49 individual dipeptides that can be encoded by the core motifs (CCAT or ATGG) of the YY1 binding consensus sequence, and determined if any of these dipeptides were over-represented in the protein sequences of the OLFR genes (Figure 3). We first calculated the frequencies of all 400 possible combinations of dipeptides using all other protein sequences except OLFR genes of the mouse to derive a reference set of the expected frequencies for the 400 dipeptides. In parallel, we also separately calculated the frequencies of the 400 dipeptides, including the 49 dipeptides that can encode the YY1 core motif, using only the protein sequences of the mouse OLFR genes. The frequencies derived from the OLFR genes (Observed Values) were compared with the reference set (Expected Values) via the Z-score to identify any over-represented dipeptides within the OLFR genes (Figure 4a) (Table 1). This global comparison did not immediately identify any dipeptides that are unusually enriched within the protein sequences of the OLFR genes. However, according to the detailed analyses using the Z-score values, two dipeptides (PL and AI) among the 49 dipeptides for the YY1 core motif showed significant enrichment (P < 0.01, Figure 4b). Even so, this enrichment is limited to the 4 bp YY1 core binding site and not the complete 10 bp motif which we used to calculate YY1 density in OLFR genes. Furthermore, the reverse-translated sequences of these two peptides (PL and AI) have 24 and 12 fold degeneracy, respectively. This means that only one out of 24 PL or 12 AI dipeptides may contain the actual core motif of the YY1 binding consensus sequence. Therefore, the detection of 4 to 8 YY1 binding motifs per one OLFR gene cannot be simply accounted for by the serendipitous overlap between the YY1 core motif and a subset of codon combinations encoding frequent peptide motifs of the OLFR genes.

**Table 1:** Dipeptide frequency in olfactory receptor proteins*

Dipeptide	All proteins	Olfactory (Observed)	Olfactory (Expected)	Frequency	Z-score
PL	77943	3222	1894	1.70	3.72
AI	41587	2784	1010	2.76	3.01
TI	35087	2444	852	2.87	2.46
SI	48732	2110	1184	1.78	1.92
PM	15764	1971	383	5.15	1.69
LH	42086	1961	1022	1.92	1.68
MA	30513	1817	741	2.45	1.44
SH	34455	1798	837	2.15	1.41
YG	25865	1130	628	1.80	0.33
AM	22983	1120	558	2.01	0.31
PF	29419	1065	715	1.49	0.23
MV	22565	939	548	1.71	0.02
SM	26009	926	632	1.47	0.00
TM	17689	786	430	1.83	-0.23
MG	19991	746	486	1.54	-0.29
PI	28078	689	682	1.01	-0.38
NH	14111	661	343	1.93	-0.43
IH	21733	609	528	1.15	-0.51
SW	17376	478	422	1.13	-0.73
MD	18778	475	456	1.04	-0.73
CH	10586	330	257	1.28	-0.97

*Only the 21/49 YY1 core forming dipeptides where observed count in olfactory receptor genes is greater than expected are shown.

**Figure 5:**
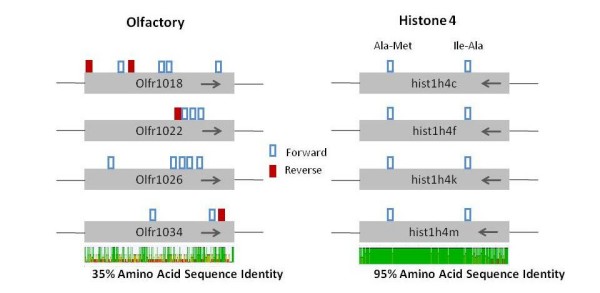
**YY1 binding sites within the ORFs of the olfactory receptor and histone 4 gene families**. Four examples of olfactory receptor genes and histone 4 genes are shown with the location and direction of their YY1 binding sites. Large gray boxes indicate open reading frames (ORFs) of these single exon genes. Boxes above each ORF show forward direction (empty box) and reverse direction (filled box) of the YY1 binding sites. The arrows in each ORF represent transcriptional directions. The bottom plot shows amino acid sequence identity for all members of each family. A total of 1,178 olfactory receptor proteins have 35% sequence identity (the four shown have 52%) while six genes of the histone 4 family share 95% identity. Conservation of position of YY1 binding sites is non-existent in olfactory genes while it is perfectly preserved in the histone 4 gene family. Directional bias in olfactory genes reflected in the figure is consistent with the complete dataset which yields 3.4 forward sites for every reverse site per 1 kb. The dipeptides encoded by the core motif of the YY1 binding site are identical for all six homologs of the histone 4 gene family (four shown). Olfactory receptor genes, by contrast, have a high number of YY1 binding sites that do not correlate to the position of these dipeptides.

As a second measure, we carefully analyzed the positions of the identified YY1 binding motifs within the ORFs of the OLFR genes (Figure 5). Conservation in location of YY1 binding site would indicate conservation of the encoded dipeptide motif whereas a lack of conservation in motif location is consistent with selection for the presence of the motif at the DNA level. The full length YY1 binding motifs within the OLFR genes do not show any patterns in their relative positions to the protein sequences of the OLFR genes as would be detected by conserved dipeptide motifs. However, there is a bias in the orientation YY1 binding sites in OLFR genes. OLFR genes contain an average of 3.4 forward sites for every reverse site per 1 kilobase. Non-OLFR genes show only a slight bias of 1.2 reverse sites for every forward site per 1 kilobase. In contrast, a similar analysis on the members of the histone 4 gene family (Hist1 h4) resulted in a different outcome. Two potential YY1 binding motifs were found within the protein-coding regions of this gene family, but the relative positions and orientations of these two motifs are identical and fixed among all the members of this gene family in mouse. The two identified YY1 binding motifs also coincide with two conserved peptide motifs (AM, and IA). Given the high levels of sequence conservation detected within the members of the histone 4 family (95% sequence identity), it is uncertain whether the identified YY1 binding motifs are genuine *cis*-regulatory elements or simply reflecting the serendipitous sequence match between the YY1 core motifs and some combinations of codons encoding conserved peptides. This pattern is in stark contrast to the random patterns associated with the position of YY1 binding motifs in the OLFR genes. This random pattern supports our initial idea that the clusters of the YY1 binding motifs within the OLFR genes most likely have been formed and maintained as *cis*-regulatory elements by purifying selection at the DNA sequence level. The bias favoring forward orientation may be an indication that forward sites enhance the efficiency of YY1 suppression of olfactory receptor genes. It is conceivable that multiple YY1 binding sites have been selected in OLFR genes with a preference for directionality as well as number. Alternatively, the non-random placement and orientation in histone 4 genes along with their extreme conservation prohibits a determination of whether selection is occurring at the DNA level or the protein level.

## Discussion

In the current study, we have shown an unusual enrichment of YY1 DNA-binding motifs in the OLFR gene family of mammals. About half of the members of the OLFR gene family have a range of 4-8 YY1 binding motifs within their protein-coding regions (Figures 1&2). Statistical analyses further confirmed that this enrichment of YY1 binding motifs is consistent with functional relevance and has likely been driven by unknown selection pressure at the DNA level. Overall, the current study suggests a potential role of YY1 or YY1-related transcription factors in the regulation of the mammalian OLFR genes. Also, this study provides further evidence that the protein-coding regions of vertebrate genes can both encode codon information and contain *cis*-regulatory elements for transcription factor binding.

The mammalian OLFR genes are expressed primarily in olfactory neurons, and only one single copy (allele) out of the entire 1,000 family members is expressed and functional in a given neuron cell [20]. This highly tissue-specific expression pattern of the OLFR genes necessitates a global repression mechanism for the majority of the OLFR genes in neural cells and in the other cell types. This mechanism acts prior to, and is separate from the negative feedback which prevents bi- and multi-allelic expression in olfactory neurons [29,30]. Since unknown mechanisms are believed to repress a large number of the OLFR genes all the time in most of the cell types, it is likely that these repression mechanisms are mediated through epigenetic modifications. Since YY1 is a well-known epigenetic regulator in the animal genome, it is plausible to propose that the identified YY1 binding motifs may play some roles in the predicted repression mechanisms for the OLFR genes [3]. In that regard, it is important to note that YY1 is one of the Polycomb group Gene (PcG) members found in both invertebrates and vertebrates [2]. Furthermore, recent studies hinted at the possibility that PcG-mediated repression mechanisms might be involved in the regulation of the OLFR genes [31]. In mutant mouse embryonic stem cells lacking the embryonic ectoderm development (EED) protein, some OLFR genes do not replicate asynchronously as they differentiate, suggesting a loss of the typical pattern of monoallelically expressed genes [32]. According to the results from another recent study, some *cis*-regulatory elements responsible for selecting one active OLFR copy in a given neuron cell are predicted to be located within the protein-coding regions of the OLFR genes [22]. This is intriguing and consistent with the observation of the current study in that some critical *cis*-elements are located within the protein-coding regions of the OLFR genes. The genetic code is optimal for containing multiple layers of encoded information within protein-coding regions [33]. In sum, although further investigation is warranted, it is likely that the identified YY1 binding motifs are functionally important *cis*-regulatory elements for the regulation of the OLFR genes.

The YY1 binding motifs identified within the OLFR genes are unusual since they are localized within the protein-coding regions of these genes and are present at high density. This is quite different from the typical pattern in which transcription factor binding sites (*cis*-regulatory elements) are located in the genomic regions surrounding the protein-coding regions of genes. OLFR genes give a fitness advantage by duplication and divergence while remaining active, yet must also retain regulatory information. According to our statistical analyses (Figure 3&4), the identified YY1 binding motifs within the OLFR genes likely represent evolutionarily selected *cis*-regulatory elements. Previous studies have shown high levels of YY1 binding affinity to the type of YY1 binding motifs found within OLFRs [1]. Though the functionality of the identified YY1 binding motifs remains to be demonstrated in vivo, it is plausible that some functional constraints serve to maintain the YY1 binding motifs within the protein-coding regions of the OLFR genes. In one scenario these motifs might be linked to the sudden expansion of this gene family in mammalian genomes. The copy number of the OLFR genes has increased dramatically in recent evolutionary times in mammalian genomes, providing a large number of receptor proteins for airborne scents [13]. The clustering of the OLFR genes in chromosomal regions also suggests this gene family may have been duplicated through *in situ *tandem duplications [13]. Tandem duplication is known to be the most frequent mechanism in increasing copy numbers for gene families [32]. However, it is not well understood how this mechanism carries over the proper information for the transcriptional regulation of duplicated gene copies. In the case of the mammalian OLFR genes, their protein-coding regions may have both information for codon and *cis*-regulatory elements so that the duplication of these genes would most likely guarantee their coding potential as well as associated transcriptional control. This might have been one functional constraint for the co-evolution of the YY1 binding motifs within the protein-coding potential of the OLFR genes.

## Conclusion

The current study reports that an unusual enrichment of YY1 binding sites, 4-8 binding sites per gene, are located in the coding regions of olfactory receptor genes in mammals. According to statistical analyses, these YY1 binding sites most likely have been selected as *cis*-regulatory elements. Also, similar patterns are found in other mammals, but not in fish, suggesting a mammalian-specific phenomenon. This study further suggests YY1 or YY1-related transcription factors as regulators of mammalian OLFR genes.

## Methods

### Visualization of YY1 binding sites in coding regions

A custom Perl script, matrix-bidirectional.pl, was run against mouse chromosome files available from Ensemble (version NCBIM37.49) ftp://ftp.ensembl.org/pub/current_fasta/mus_musculus/dna/ (see Additional file 1) [34]. The program calculates score by matching a 10 bp window to a matrix of the likelihood for each position (Additional files 2 &3) along the 10 bp consensus sequence (the Position Weight Matrix, PWM). Each base pair is given a value equivalent to the decimal percentage of its match to the known YY1 binding motifs. The four base pair core, CCAT, is scored at 100% for each base plus 2. Flanking bases vary in score from 0 to 1 based upon their frequency in known YY1 binding motifs. The total score for each 10 bp window is calculated and compared to our cutoff score of 8.0 which indicates a good match. Our previous work revealed that scores above 8.0 correlate with good YY1 binding *in vitro *[1,35]. Output was generated in the WIG format for each chromosome with each YY1 binding motif score represented by start and end position and bar height corresponding to the PWM score match. Position weight matrix scores and location information were loaded into the University of California, Santa Cruz (UCSC) Genome Browser for visualization of YY1 location (Figure 1) [36].

### Motif finding and scoring

Our Perl script was run against the mouse, human and zebrafish mRNA available from NCBI ftp://ftp.ncbi.nih.gov/genomes/. Each gene was scored for number and quality of YY1 motifs. Results were sorted by a YY1 density score, the combined score of YY1 motifs divided by the length of the gene. Predicted and hypothetical genes were removed from the mouse yielding 40,009 total genes (19,818 removed, 20,191 remaining), with 19,083 non-olfactory and 1,108 olfactory genes. Hypothetical genes were removed from the human transcriptome, yielding 24,886 total genes with 24,506 non-olfactory and 380 olfactory genes. Hypothetical genes were removed from the zebrafish transcriptome, yielding 9,092 genes with 9,067 non-odorant genes and 25 odorant receptors. We removed the hypothetical and predicted genes because they may not contain complete ORFs. The upstream 1 kb regions of 20419 Refseq genes in the mouse was obtained from UCSC http://hgdownload.cse.ucsc.edu/downloads.html.

Histograms were made in Microsoft Excel 2007 by sorting the genes by YY1 density from high to low, then assigning a count to order the genes. Olfactory receptor genes were separated from non-olfactory receptor genes and the count numbers were used as position information to make a histogram which shows the distribution of OLFRs along the range of YY1 containing genes (Figure 2).

### Protein motif correlation testing

The mouse proteome was downloaded from NCBI ftp://ftp.ncbi.nih.gov/genomes/M_musculus/protein/protein.fa.gz which contains 34,966 peptide sequences and nomenclature. Olfactory receptor proteins (1,178) were separated from non-olfactory proteins (33,788). A Perl script, dipep-singlefile.pl was used to generate a count of each of the 400 possible dipeptides in each of these groups (see Additional file 4).

A Z-test was performed according to the formula Z = (observed - μ)/δ where observed is the count of each possible dipeptide found in olfactory receptors, μ is the mean of the counts from the whole population of non-olfactory proteins, and δ is the standard deviation of the population count. Z-score units are given in standard deviations from the mean. Expected count was calculated by multiplying the frequency of each dipeptide from all non-olfactory receptor proteins by the total number of dipeptides seen in OLFR proteins. Figure 4 shows the plot generated in Microsoft Excel 2007 comparing the observed to expected ratio for all 400 possible dipeptides and the subset of 49 YY1 core-forming dipeptides which exhibits no difference in the distribution of the subset.

We found 21 of the 49 dipeptides which can make up a YY1 binding site had greater than expected values, but only 2 were over 3 standard deviations away from the mean (Table 1, Figure 4). Table 1 shows only the dipeptides in which the observed count in olfactory genes was higher than the expected count in mouse.

Global alignment was done using ClustalW with 1,178 OLFR and 6 hist1 h4 amino acid sequences from mouse (Figure 5) [37]. All Perl scripts are available for download on our website at http://JooKimLab.lsu.edu[38].

## Abbreviations

YY1: Yin-Yang 1; SINE: Short interspersed element; LINE: Long interspersed element; OLFR: Olfactory receptor; OR: Odorant receptor; PWM: Position weight matrix; UCSC: University of California at Santa Cruz; ORF: Open reading frame; WIG: Wiggle format; NCBI: National center for biotechnology information.

## Authors' contributions

CF carried out the coding, genome scanning, performed the statistical analyses, and participated in the design of the study. JK conceived of the study, and participated in its design and coordination. All authors read and approved of the final manuscript.

## Supplementary Material

Additional file 1 **Position Weight Matrix Perl program**. Perl program "matrix-bidirectional.pl.txt" uses position weight matrix files and fasta formatted input files to generate positional and scoring data for YY1 binding sites. Click here for file

Additional file 2 **YY1 forward matrix**. Position weight matrix file for "matrix-bidirectional.pl.txt" named "YY1_F.matrix". Click here for file

Additional file 3 **YY1 reverse matrix**. Position weight matrix file for "matrix-bidirectional.pl.txt" named "YY1_R.matrix". Click here for file

Additional file 4 **Dipeptide count Perl program**. Perl program "dipep-singlefile.txt.pl" requires fasta formatted input files to generate dipeptide counts. Click here for file

## References

[B1] Kim JD Faulk C Kim J Retroposition and evolution of the DNA-binding motifs of YY1, YY2 and REX1 Nucleic Acids Res 2007 35 10 3442 3452 10.1093/nar/gkm235 17478514 PMC1904287

[B2] Shi Y Lee JS Galvin KM Everything you have ever wanted to know about Yin Yang 1 Biochim Biophys Acta 1997 1332 2 F49 F66 9141463 10.1016/s0304-419x(96)00044-3

[B3] Gordon S Akopyan G Garban H Bonavida B Transcription factor YY1: structure, function, and therapeutic implications in cancer biology Oncogene 2006 25 8 1125 1142 10.1038/sj.onc.1209080 16314846

[B4] Thomas MJ Seto E Unlocking the mechanisms of transcription factor YY1: are chromatin modifying enzymes the key? Gene 1999 236 2 197 208 10.1016/S0378-1119(99)00261-9 10452940

[B5] Wilkinson FH Park K Atchison ML Polycomb recruitment to DNA in vivo by the YY1 REPO domain Proc Natl Acad Sci USA 2006 103 51 19296 19301 10.1073/pnas.0603564103 17158804 PMC1748220

[B6] Schug J Schuller WP Kappen C Salbaum JM Bucan M Stoeckert CJ Promoter features related to tissue specificity as measured by Shannon entropy Genome Biol 2005 6 4 R33 10.1186/gb-2005-6-4-r33 15833120 PMC1088961

[B7] Sui G Affar el B Shi Y Brignone C Wall NR Yin P Donohoe M Luke MP Calvo D Grossman SR Shi Y Yin Yang 1 is a negative regulator of p53 Cell 2004 117 7 859 872 10.1016/j.cell.2004.06.004 15210108

[B8] Satyamoorthy K Park K Atchison ML Howe CC The intracisternal A-particle upstream element interacts with transcription factor YY1 to activate transcription: pleiotropic effects of YY1 on distinct DNA promoter elements Mol Cell Biol 1993 13 11 6621 6628 8413258 10.1128/mcb.13.11.6621-6628.1993 PMC364725

[B9] Humphrey GW Englander EW Howard BH Specific binding sites for a pol III transcriptional repressor and pol II transcription factor YY1 within the internucleosomal spacer region in primate Alu repetitive elements Gene Expr 1996 6 3 151 168 9041122 PMC6148310

[B10] Buck L Axel R A novel multigene family may encode odorant receptors: a molecular basis for odor recognition Cell 1991 65 1 175 187 10.1016/0092-8674(91)90418-X 1840504

[B11] Zozulya S Echeverri F Nguyen T The human olfactory receptor repertoire Genome Biol 2001 2 6 research0018.1 research0018.12 10.1186/gb-2001-2-6-research0018 PMC33394 11423007

[B12] Zhang X Rodriguez I Mombaerts P Firestein S Odorant and vomeronasal receptor genes in two mouse genome assemblies Genomics 2004 83 5 802 811 10.1016/j.ygeno.2003.10.009 15081110

[B13] Niimura Y Nei M Evolutionary dynamics of olfactory and other chemosensory receptor genes in vertebrates J Hum Genet 2006 51 6 505 517 10.1007/s10038-006-0391-8 16607462 PMC1850483

[B14] Alioto TS Ngai J The odorant receptor repertoire of teleost fish BMC Genomics 2005 6 173 10.1186/1471-2164-6-173 16332259 PMC1325023

[B15] Kambere MB Lane RP Co-regulation of a large and rapidly evolving repertoire of odorant receptor genes BMC Neurosci 2007 8 Suppl 3 S2 10.1186/1471-2202-8-S3-S2 17903278 PMC1995454

[B16] Keller A Vosshall LB Better smelling through genetics: mammalian odor perception Curr Opin Neurobiol 2008 18 4 364 369 10.1016/j.conb.2008.09.020 18938244 PMC2590501

[B17] Feldmesser E Olender T Khen M Yanai I Ophir R Lancet D Widespread ectopic expression of olfactory receptor genes BMC Genomics 2006 7 121 10.1186/1471-2164-7-121 16716209 PMC1508154

[B18] Spehr M Schwane K Riffell JA Zimmer RK Hatt H Odorant receptors and olfactory-like signaling mechanisms in mammalian sperm Mol Cell Endocrinol 2006 250 128 136 10.1016/j.mce.2005.12.035 16413109

[B19] Pluznick JL Dong-Jing Z Xiaohong Z Qingshang Y Rodriguez-Gil DJ Eisner C Wells E Greer CA Wang T Firestein S Schnermann J Caplan MJ Functional expression of the olfactory signaling system in the kidney Proc Natl Acad Sci USA 2009 106 6 2059 2064 10.1073/pnas.0812859106 19174512 PMC2644163

[B20] Chess A Simon I Cedar H Axel R Allelic inactivation regulates olfactory receptor gene expression Cell 1994 78 5 823 834 10.1016/S0092-8674(94)90562-2 8087849

[B21] Shykind BM Regulation of odorant receptors: one allele at a time Hum Mol Genet 2005 14 Spec No 1 R33 R39 10.1093/hmg/ddi105 15809271

[B22] Nguyen MQ Zhishang Z Marks CA Ryba NJP Belluscio L Prominent roles for odorant receptor coding sequences in allelic exclusion Cell 2007 131 5 1009 1017 10.1016/j.cell.2007.10.050 18045541 PMC2195930

[B23] Merriam LC Chess A cis-Regulatory elements within the odorant receptor coding region Cell 2007 131 5 844 846 10.1016/j.cell.2007.11.016 18045531

[B24] Kim J YY1's longer DNA-binding motifs Genomics 2009 93 2 152 158 10.1016/j.ygeno.2008.09.013 18950698 PMC2668202

[B25] Kang K Chung JH Kim J Evolutionary Conserved Motif Finder (ECMFinder) for genome-wide identification of clustered YY1- and CTCF-binding sites Nucleic Acids Res 2009 37 6 2003 2013 10.1093/nar/gkp077 19208640 PMC2665242

[B26] Kim JD Hinz AK Bergmann A Huang JM Ovcharenko I Stubbs L Kim J Identification of clustered YY1 binding sites in imprinting control regions Genome Res 2006 16 7 901 911 10.1101/gr.5091406 16760423 PMC1484457

[B27] Smit AFA Hubley R Green P RepeatMasker Open-3.0 1996

[B28] UCSC Genome Browser http://genome.ucsc.edu/

[B29] Lewcock JW Reed RR A feedback mechanism regulates monoallelic odorant receptor expression Proc Natl Acad Sci USA 2004 101 4 1069 1074 10.1073/pnas.0307986100 14732684 PMC327152

[B30] Serizawa S Miyamichi K Sakano H Negative feedback regulation ensures the one neuron-one receptor rule in the mouse olfactory system Chem Senses 2005 30 Suppl 1 i99 100 10.1093/chemse/bjh133 15738216

[B31] Alexander MK Mlynarczyk-Evans S Royce-Tolland M Plocik A Kalantry S Magnuson T Panning B Differences between homologous alleles of olfactory receptor genes require the Polycomb Group protein Eed J Cell Biol 2007 179 2 269 276 10.1083/jcb.200706053 17954609 PMC2064763

[B32] Ohno S Evolution by gene duplication 1970 London, NY: Allen & Unwin; Springer-Verlag

[B33] Itzkovitz S Alon U The genetic code is nearly optimal for allowing additional information within protein-coding sequences Genome Res 2007 17 4 405 412 10.1101/gr.5987307 17293451 PMC1832087

[B34] Flicek P Ensembl 2008 Nucleic Acids Res 2008 36 D707 D714 10.1093/nar/gkm988 18000006 PMC2238821

[B35] Kim J Multiple YY1 and CTCF binding sites in imprinting control regions Epigenetics 2008 3 3 115 118 18458536 10.4161/epi.3.3.6176

[B36] Kent WJ Sugnet CW Furey TS Roskin KM Pringle TH Zahler AM Haussler D The human genome browser at UCSC Genome Res 2002 12 6 996 1006 12045153 10.1101/gr.229102 PMC186604

[B37] Larkin MA Blackshields G Brown NP Chenna R McGettigan PA McWilliam H Valentin F Wallace IM Wilm A Lopez R Thompson JD Gibson TJ Higgins DG Clustal W and Clustal X version 2.0 Bioinformatics 2007 23 21 2947 2948 10.1093/bioinformatics/btm404 17846036

[B38] The Joomyeong Kim Lab Website http://JooKimLab.lsu.edu

